# A promising alternative to opioids

**DOI:** 10.7554/eLife.103003

**Published:** 2024-09-30

**Authors:** Jie Zhang, Jianguo Cheng

**Affiliations:** 1 Department of Pain Management, Cleveland Clinic Cleveland United States; 2 Department of Neuroscience, Cleveland Clinic Cleveland United States

**Keywords:** opioids, Post-surgical pain, clarix FLO, HC-HA/PTX3, amniotic membrane, Mouse

## Abstract

A complex extracted from the amniotic membrane in humans reduces post-surgical pain in mice by directly inhibiting pain-sensing neurons.

**Related research article** Zhang C, Huang Q, Ford NC, Limjunyawong N, Lin Q, Yang F, Cui X, Uniyal A, Liu J, Mahabole M, He H, Wang XW, Duff I, Wang Y, Wan J, Zhu G, Raja SN, Jia H, Yang D, Dong X, Cao X, Tseng SC, He SQ, Guan Y. 2024. Human birth tissue products as a non-opioid medicine to inhibit post-surgical pain. *eLife* **13**:RP101269. doi: 10.7554/eLife.101269.

Post-surgical pain affects millions of people every year, and managing it is a critical aspect of patient care (Tait et al., 2018). Effective pain relief is essential both for comfort and also for preventing complications such as chronic pain or delayed recovery.

Traditionally, a broad group of pain-relieving medicines known as opioids have been the cornerstone of post-surgical pain treatment. By binding to pain receptors, opioids reduce pain intensity. However, opioids can cause nausea, constipation and respiratory depression, and they also have the potential to be addictive (Stein, 2016). Indeed, their widespread use is believed to have contributed to an opioid epidemic that has resulted in high rates of addiction, overdose and death, particularly in the United States (Hornberger and Chhatwal, 2021; The Lancet., 2021). This underscores the need for alternative pain management strategies that can effectively control pain without causing dangerous side effects.

For decades, the amniotic membrane – the innermost layer of the placenta – has been used to heal wounds and to repair damage to the surface of the eye through its anti-inflammatory and anti-scarring properties (Díaz-Prado et al., 2011; Law et al., 2022). Now, in eLife, Yun Guan and Shao-Qui He of Johns Hopkins University and colleagues – including Chi Zhang, Qian Huang, and Neil Ford as joint first authors – report that a human amniotic membrane product shows promise as an opioid alternative for post-surgical pain management (Zhang et al., 2024).

Clarix Flo (or FLO for short) contains a rich matrix of biologically active molecules derived from the amniotic membrane that can modulate cellular activity. To investigate whether FLO can reduce post-surgical pain, Zhang et al. applied it to surgical sites in mice, finding that this significantly reduced sensitivity to post-surgical pain. This effect was shown to depend on CD44, a cell surface receptor that is involved in various physiological and pathological processes. By interacting with the CD44 receptor, FLO inhibits the activity of specialized sensory neurons located in the dorsal root ganglia that are responsible for transmitting pain signals to the central nervous system. This means that FLO targets pain signaling at its source, which is markedly different from how opioids work.

To identify the component within FLO responsible for this effect, the team isolated a complex known as HC-HA/PTX3, which is found in uniquely high amounts in birth tissues. Applying this complex alone replicated the pain-inhibiting effects of FLO. HC-HA/PTX3 was also purer than FLO and more soluble in water, which increases its therapeutic potential by making it less likely to cause adverse effects and more likely to reach its target site. Further experiments revealed that HC-HA/PTX3 induces cytoskeletal rearrangements in pain-sensing neurons. This inhibits critical sodium and high-voltage calcium currents that are vital for propagating pain signals, significantly reducing the ability of these neurons to transmit pain signals to the central nervous system (Figure 1).

**Figure 1.**
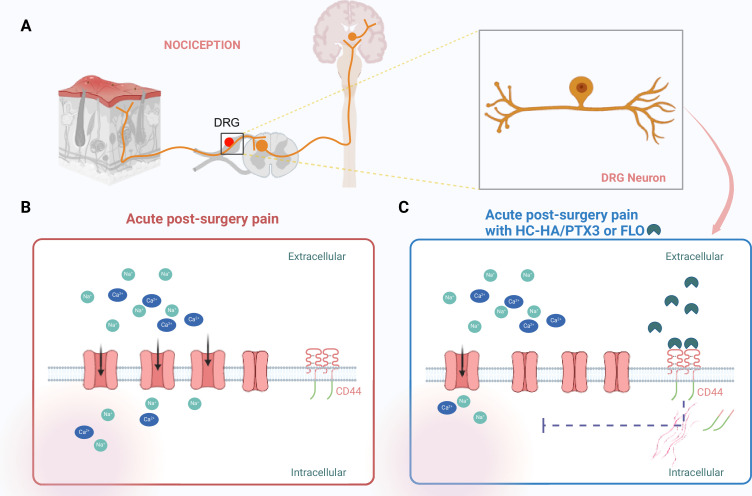
Targeting pain signaling at its source. (**A**) Painful stimuli are detected through a process called nociception. Neurons (orange) located in the dorsal root ganglia (DRG) relay pain signals from a peripheral wound (such as a skin injury; left) to the central nervous system (right), which results in pain being experienced. (**B**) After surgery, sodium ions (Na^+^; green) and calcium ions (Ca^2+^; blue) flow into DRG nociceptive neurons through ion channels (pink) that are embedded in the cell wall of the neurons. This contributes to acute post-surgical pain. (**C**) HC-HA/PTX3 or FLO (dark green shapes) can bind to CD44 receptors (red lines) on the surface of the DRG neurons. This binding leads to a rearrangement of the cytoskeleton within the cell, which pushes the receptors into the neuron, causing some of the ion channels to close. The resulting reduction in the influx of sodium and calcium ions leads to a decrease in pain signaling. This figure was created using BioRender.com.

The discovery that HC-HA/PTX3 is the key bioactive component in FLO makes it a potential candidate for acute post-surgical and chronic pain management in various clinical settings. While this opens exciting avenues for future research, before HC-HA/PTX3 can be fully translated from preclinical research to clinical application, important questions must be answered. One key challenge is determining whether the effects observed in mice translate to human patients. Although pain signaling pathways are largely conserved across species, human clinical trials are necessary to confirm the efficacy and safety of HC-HA/PTX3. Researchers are also considering whether combining the complex with other non-opioid treatments, such as anti-inflammatory drugs or nerve growth inhibitors, could create a more comprehensive approach to pain management.

Despite these uncertainties, the findings of Zhang et al. represent a significant step forward in the search for effective, non-opioid pain therapies. By targeting the underlying pain mechanisms at the cellular level, rather than simply masking the symptoms as opioids do, biologically derived products like HC-HA/PTX3 could revolutionize post-surgical and chronic pain treatment. While much work remains to bring these discoveries to clinical practice, the promise of safer, more effective pain management is an exciting prospect in the ongoing fight against the opioid epidemic.
